# Some Phases of Malaria

**Published:** 1915-06

**Authors:** R. H. Crockett


### Some Phases of Malaria.

BY R. H. CROCKETT, M. D.

### OCCURRENCE.

There is quite a variance of opinion, in both the medical and lay mind, as to the prevalence of malaria. The writer knows, from personal experience, that malaria has been of frequent occurrence, in Central, South, and Southeast Texas.

Many doctors of Central Texas will say that malaria is almost rare in their section. The trouble is, that the doctor is not called in for malaria, quite often; and thus overlooks its existence. The writer recalls many quarts of balmoric infusion; and the death of his childhood friend from a "so-called" congestive chill, that occurred in Central Texas.

In the hospitals of Galveston and Houston malaria is quite common, especially if it is a wet year; and wet years are not unusual in those sections. It is true that some of the cases drift in from Mexico and Louisiana; but many of them come from the country in the near vicinity. At St. Joseph's Infirmary, Houston, Texas, during the months of October, November, December and January, the writer demonstrated the malarial organisms in blood from fifteen patients that he treated, and also found the organisms in the blood of several patients for other doctors. Five of these cases came from Louisiana and Mexico, seven from East Texas, and three cases from Houston. If there were fifteen cases in one hospital in four months, how many were there outside? And yet, one of the oldest doctors of Houston said malaria was a rare disease in that section.

### DIAGNOSIS.

A very prevalent opinion is that anyone can diagnose malaria from a short history, and never make a mistake. Many cases of malaria are diagnosed as typhoid fever, yellow jaundice, congestive chills, fits, poisoning, etc. On the other hand, many cases of intestinal toxemia, typhoid fever, pyemia, foetid bronchitis, etc., are diagnosed as malaria. These errors of diagnosis come from the fact that we try to diagnose malaria from the "chill and fever" idea, instead of going through a careful bedside and laboratory routine.

The main error of diagnosis, as malaria, when the case is otherwise, is due to the history of a chill. Rigors or chills are quite common in typhoid fever, any pus focus, and many other troubles. The failure to diagnose malaria is often due to the irregular symptoms; the lack of a microscope, or poor laboratory technique.

The microscope might lead one to an occasional doubtful diagnosis, on the negative side; but the microscope, if correctly used, is by far the strongest factor in any obscure case of malaria. If repeated blood stains do not show the malarial organism one had best look very carefully for some other cause before it is too late.

In making a blood stain, the following points should be observed: (1) Wash the slide clean in alcohol 95 per cent; pass it through a flame, touching both sides till quite warm and keep free from all dust or grit; (2) do not use too large a drop of blood; (3) if using the commercial tablets, dilute with about two or three times as much alcohol as the directions call for, as the organisms are often hidden by the overstained corpuscle.

### TREATMENT.

In the treatment of malaria, the writer has been rather a close follower of the ideas advanced by Dr. Bass, of the department of tropical medicine, Tulane University.

As soon as the case is diagnosed, the patient, if an adult, is given thirty grains of quinine, by mouth. The quinine may be given in capsule, but the best way is to dissolve it in warm (or cold) water, by adding a few drops of dilute hydrochloric (muriatic) acid (or a little vinegar). The second day, give three doses of ten grains each, and this may just as well be given in capsule, if the fever has dropped. Give thirty grains a day for seven days, and then only give quinine every seventh day, thirty grains, for two months longer. If the patient is anemic, give the necessary iron, etc. Give babies and children doses in proportion to their body weight. The average adult weighs about 150 pounds. A child of 15 pounds should get three grains of quinine a day, and one of 10 pounds, two grains a day.

Many contend that hypodermic use of quinine is better; and others that the dose is too large. In the fifteen cases that I treated, there were no apparent evil effects from the large dosage by mouth. One case came in unconscious and almost dead. We gave him quinine hypodermically, but he died before the quinine could take effect. Three other cases were very delirious, but responded promptly to the quinine by mouth. In the fourteen cases, the temperature fell to normal in twenty-four hours or sooner, and did not rise again while in the hospital. One case, coming in with very severe mental symptoms, responded promptly to the treatment, but some mental symptoms returned on the seventh day. The quinine was stopped; the mental symptoms disappeared, and the patient stayed at the hospital quite a while, with no return of symptoms.

### REPORT OF A CASE.

Mr. A, male, white, age 21, brought into hospital wildly delirious, with constant outcry. History—walked into a building, and fell to the floor. The patient's face seemed quite hyperemic, but other physical signs were normal, except the pupils were quite dilated and would not respond to light, and he resisted all movement. Preliminary diagnoses of poisoning, hysteria and uremia were made. A large quantity of food was withdrawn from the stomach, but the food seemed to indicate nothing. Catheterization showed a considerable quantity of normal urine. Two-tenths grains of apomorphine had not the slightest effect. A blood count was negative.

The patient was tied in bed and kept under careful observation. He continued to make a loud whining noise and to thresh his limbs about for twenty-four hours, and then gradually regained consciousness. About twelve hours after entering the hospital he was untied. He got a little quieter and did not fall out of the bed, but continued a loud noise and moderate threshing movements for twenty-four hours, until he began to regain complete consciousness.

His brother came in some twelve hours after Mr. A. entered the hospital and said Mr. A. had been out of the Sealy Hospital at Galveston for about a week, where he had been treated for malaria, and that Mr. A. had taken a little quinine every day since; hence our failure to find the organism in the blood. When Mr. A. regained complete consciousness his pupils became normal. He told me that he was taken to the Galveston hospital almost unconscious, and treated for malaria two weeks, at first being given twenty-five grains a day of quinine, hypodermically. He thought he had been well for a week. He felt well and wanted to go home.

We kept him another day. His temperature began to rise about forty-eight hours after he first came in, and his pupils began to dilate again. His blood was re-examined and one stippled cell found. I gave him thirty grains of quinine by mouth at once. His temperature fell to normal, and his pupils became normal in about eight hours. He was given thirty grains of quinine each day, and remained normal until he left the hospital. He was instructed to follow the treatment as above outlined.

